# AN INTERGENERATIONAL DYADIC APPROACH TO UNDERSTANDING GRANDPARENT DEATH WITHIN A FAMILY SYSTEM

**DOI:** 10.1093/geroni/igz038.1039

**Published:** 2019-11-08

**Authors:** Margaret M Manoogian, Heidi Igarashi, Maggie Leinenweber

**Affiliations:** 1 Western Oregon University, Monmouth, Oregon, United States; 2 Oregon State University, Corvallis, Oregon, United States

## Abstract

Due to increased longevity and generational location, grandparent death creates new contexts for identity, family culture, and intergenerational relationships. To explore this loss from two perspectives, we conducted intensive interviews with young adults and their mothers (N = 16) who experienced a recent grandparent (parent) death. Guided by the life course perspective, we were interested to learn how grandparent death may shape identity, meaning, and behaviors among family members, and influence their shared parent-child relationship. Findings suggest that the relationship with the grandparent, the diverse expressions of grief, the navigation of family transitions during and after death, and the curation of grandparent memories influenced individual and family outcomes. Implications suggest the need for varied supports that are sensitive to how individual family members approach grief in distinct ways reflective of their developmental positions, past experiences, and relational expectations.

